# Latency to delivery and incidence of adverse obstetric and perinatal outcomes in preterm premature rupture of membranes before 32 weeks

**DOI:** 10.1007/s00404-025-07970-3

**Published:** 2025-02-10

**Authors:** Viola Seravalli, Chiara Colucci, Chiara Di Cencio, Anna Morucchio, Federica Barsanti, Mariarosaria Di Tommaso

**Affiliations:** Department of Health Sciences, Division of Obstetrics and Gynecology, University of Florence, Largo G. A. Brambilla, 3, 50134 Florence, Italy

**Keywords:** Premature rupture of membrane, Latency to delivery, Preterm birth, Neonatal survival, Placental abruption

## Abstract

**Purpose:**

The aim of this study was to evaluate the average latency to delivery, obstetric outcomes and neonatal survival in pregnancies complicated by preterm premature rupture of membranes (PPROM) before 32 weeks.

**Methods:**

A retrospective study was conducted on pregnant women admitted for PPROM before 32 weeks. Patients were categorized into three groups based on gestational age (GA) at PPROM (< 24, 24 to 28, 28 to 32 weeks). Latency to delivery, obstetric outcomes and neonatal survival were analyzed.

**Results:**

86 women who had PPROM before 32 weeks were identified. The mean GA at PPROM was 26.1 weeks and the median latency to delivery was 16 days (IQR 4, 27). The median latency to delivery was 22 days for previable PPROM, 11 days for PPROM between 24 and 28 weeks, and 16 days for PPROM between 28 and 32 weeks (p = 0.29). All cases of placental abruption (7/86, 8%) and cord prolapse (6/86, 7%) occurred in women with PPROM before 28 weeks. In 44% of PPROM, placental histology demonstrated chorionamnionitis. Neonatal survival at discharge was significantly lower in previable PPROM (< 24 weeks) compared to PPROM at 24–26 weeks (58% vs 92%, p = 0.04), and it reached 100% in cases of PPROM after 28 weeks.

**Conclusion:**

In PPROM occurring before 32 weeks the median latency to delivery ranged between 11 and 22 days. Neonatal survival improves with higher GA at PPROM, and it increases by more than 33% when PPROM occurs after 24 weeks of gestation. These data may be valuable for patient counselling.

## What does this study add to the clinical work

**Table:** 

This study provides information on maternal and neonatal outcomes of early, very-early and pre-viable pPROM, stratified by the gestational age at PPROM, enabling more personalized counselling for patients.	

## Introduction

Preterm birth is a significant public health concern worldwide, accounting for a substantial portion of neonatal morbidity and mortality [1]. The global preterm birth prevalence is 9.9%, with variations ranging from 7.8% to 13.3% depending on the country [2]. Among the numerous factors contributing to preterm birth, Preterm Premature Rupture of Membranes (PPROM) stands out as a critical precipitating event. PPROM is defined as the rupture of fetal membranes before the onset of labor at a gestational age of less than 37 weeks [3]. When PPROM happens prior to reaching the fetal viability threshold, usually before 24 weeks of gestation, it is referred to as previable PPROM (pPPROM) [4]. PPROM occurs in approximately 3% of pregnancies and is mostly responsible for, or associated with, almost one-third of preterm birth. It causes prematurity related complications in the neonate [5–7], including respiratory distress syndrome, intraventricular hemorrhage, necrotizing enterocolitis, sepsis and death [3, 8]. PPROM itself results in immediate risk of chorioamnionitis, placental abruption and cord prolapse, leading to emergency cesarean section [9–12]. A history of PPROM is also a strong risk factor for recurrence, with reported recurrence rates of 13–32% [13, 14].

The management of PPROM has evolved over the years, and it primarily depends on the gestational age at membrane rupture. The gestational age at delivery is assumed to be the most important factor in determining neonatal outcomes after PPROM. Accepted management includes, in selected cases, hospitalization and administration of intramuscular corticosteroids and antibiotics to prolong latency [15], when PPROM occurs between 24 and 34 weeks. PPROM occurring prior to 24 weeks of gestation are usually managed with home care, until the time of viability is reached [3]. Some guidelines consider the possibility of offering outpatient care to women with PPROM even after 24 weeks, following a period of in-patient care, making this decision on an individual basis [16]. The management of PPROM also depends on various factors such as presence of signs of infection, the condition of the fetus, and the occurrence of other maternal or fetal complications. In patients with PPROM before 34 + 0 weeks of gestation and with no contraindications to continuing the pregnancy, such as abnormal results from fetal testing or intrauterine infection, expectant management likely provides benefit for the woman and newborn [3, 17]. Between 34 + 0 and 36 + 6 weeks (late PPROM), the gestational age at which delivery is indicated remains controversial. Both expectant management, in the absence of signs of infection or fetal compromise, and immediate induction of labor are acceptable approaches.

Indeed, numerous studies have demonstrated the benefits of adopting expectant management for PPROM for both the mother and the baby [18, 19].

Despite its clinical importance, PPROM remains a complex and multifaceted condition, and many aspects of its pathophysiology, diagnosis, and management are still under investigation. Providing accurate counselling to patients regarding the management of PPROM and the potential adverse perinatal outcomes is crucial. Understanding the anticipated duration between PPROM and delivery, as well as the frequency of obstetric and perinatal adverse outcomes, can be a valuable tool.

The objective of our study was to assess the average latency between preterm premature rupture of membranes and delivery in PPROM occurring before 32 weeks. We also aimed to analyze the frequency of adverse obstetric outcomes and the neonatal survival rate based on the gestational age at PPROM, with the goal of improving counseling for pregnant women experiencing this pregnancy complication.

## Material and methods

This retrospective study was conducted on singleton pregnancies complicated by PPROM from January 2018 to June 2023 at Careggi Hospital in Florence, Italy. Patients were diagnosed with PPROM with a combination of signs and symptoms indicative of rupture of membranes (visualization of amniotic fluid passing from the cervical canal and pooling in the vagina), decreased amniotic fluid by ultrasonogram and detection of Insulin Growth Factor Binding Protein-1 (IGFBP-1) in vaginal fluid obtained from vaginal swab samples.

Women carrying a pessary or cerclage were excluded along with patients whose fetuses were affected by major malformation, those opting for pregnancy termination and those carrying multiple pregnancies. After collecting all the data from the hospital’s electronic medical software, the gestational age (GA) at PPROM was documented and cases of membrane ruptured occurred before 32 weeks were included. GA at delivery was collected and latency to delivery was calculated. Maternal demographic and obstetrics characteristics were recorded, and data on obstetric and neonatal outcome were collected. The study population was then categorized into five groups based on the gestational age at PPROM (< 24, 24–27^+6^, 28–31^+6^ weeks). Within each group, we assessed the latency between the occurrence of PPROM and delivery, as well as the rate of women delivering within 48 h and within 7 days from the membrane rupture. Furthermore, we investigated the occurrence of the major adverse obstetric outcomes, including placental abruption, cord prolapse, cesarean section, and chorioamnionitis (both clinically suspected and confirmed by placental histology), and we compared the incidence of such pregnancy complications between cases of very-early PPROM (< 28 weeks) and those occurring at later gestational ages. Finally, we evaluated the live births rate and the neonatal survival upon discharge.

The following protocol is used at our Institution in cases of PPROM. Pregnancy termination is offered for previable PPROM up to 22 weeks of gestation. Hospitalization is recommended once viability has been reached. The administration of a single course of corticosteroids is indicated for pregnant women with PPROM between 24 + 0 and 33 + 6 weeks of gestation who are at risk of preterm birth within 7 days. A single repeat course of antenatal corticosteroids can be considered in women with PPROM who are less than 34 weeks of gestation, are at risk of preterm delivery within 7 days, and whose prior course of antenatal corticosteroids was administered before 26 weeks’ gestation and more than 14 days previously. Tocolytic agents are typically considered in women with PPROM and regular contractions, but only to allow time for corticosteroid administration, particularly at earlier gestational ages; however, tocolytics are avoided if there is evidence of infection or placental abruption.

A 7-day course of latency antibiotics, consisting of intravenous ampicillin and azithromycin followed by oral amoxicillin and azithromycin, is recommended during the expectant management of women with PPROM between 24 and 36 weeks of gestation. Specific antibiotic treatment is administered in cases of positive vaginal and/or urinary cultures. In PPROM occurring before 24 weeks, when hospital admission is not yet indicated, a 7-day course of amoxicillin is administered at home. Antenatal CTG monitoring during hospitalization begins at 26 weeks. Delivery is indicated in the presence of infection, abnormal CTG findings, placental abruption, or cord prolapse. A non-cephalic fetal presentation is an indication to cesarean section only after 26 weeks.

### Statistical analysis

Data distribution was assessed according to the Shapiro–Wilk’s test of normality. Results were reported as absolute and relative frequencies for categorical variables and median and interquartile range for continuous variables. Analyses were performed using the Mann–Whitney U-test for continuous variables, and the Chi-Squared and Fisher’s Exact tests for categorical data. Comparisons among more than two groups were conducted using the Kruskal–Wallis test. A p-value < 0.05 was considered significant. The risk of preterm delivery in each group was also assessed using Kaplan–Meier analysis, in which gestational age was the timescale and spontaneous delivery was the event. Data analysis was performed using SPSS 20.0 (SPSS Inc, Chicago, IL).

This study was approved by the Ethics Committee of Careggi University Hospital (study ID 23388).

## Results

**Fig. 1:**
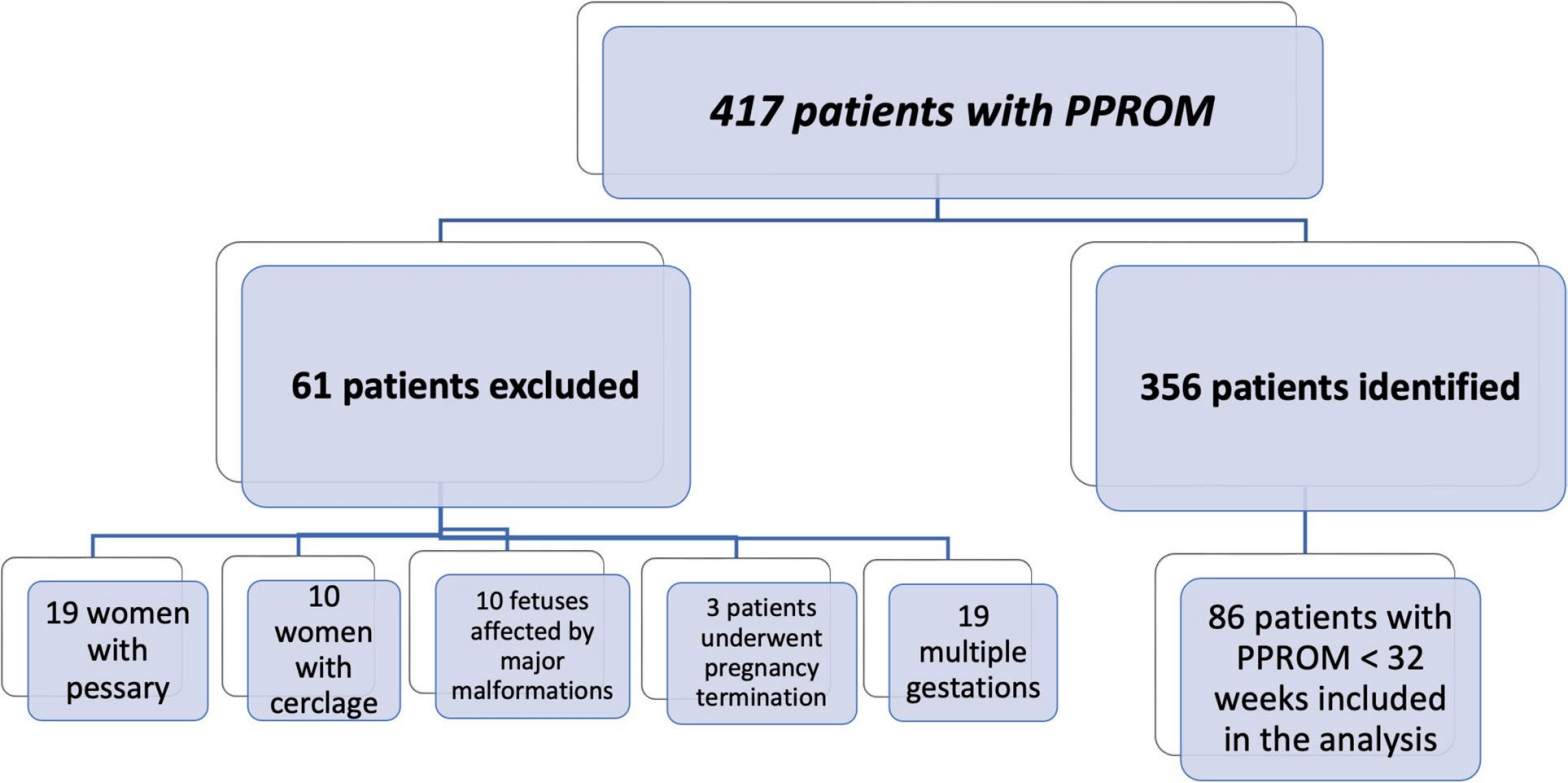
Flow chart of the women’s selection process (January 2018–June 2023)

During the study period, we identified a total of 417 women who were admitted for PPROM and subsequently delivered at our hospital, corresponding to an incidence rate of 2.5% (417/16631 total deliveries during the study period). After removing cases that met one or more of the previously defined exclusion criteria, 356 PPROM were identified, of which 86 (24%) occurred before 32 weeks and were included in the analysis (Fig. 1). The maternal characteristics of the study cohort are reported in Table 1. The mean maternal age was 34 years, most patients were of Caucasian ethnicity and had a normal BMI. About half of the women were nulliparous, and 9% of patients had a history of prior preterm birth. Table 1 also reports data on the amniotic fluid level (maximum vertical pocket) at admission, during hospital stay, and before delivery, when available, as well as the incidence of oligohydramnios. All women in our cohort received antibiotic therapy as per hospital protocol, with a combination of ampicillin and azithromycin. 50/86 women (57%) received tocolysis at some point during their hospital stay.

**Table 1:** Maternal and pregnancy characteristics of the study cohort (n = 86)

Characteristics	Data
Age (years)	34.0 (± 6.3)
BMI (kg/m^2^)	22.5 (20.8, 25.5)
Parity	
Nulliparous	45 (52.3%)
Multiparous	41 (47.7%)
Previous preterm birth (PTB)	
No history of PTB	78 (90.7%)
Previous PTB	8 (9.3%)
Smoking during pregnancy	7 (8.1%)
Ethnicity	
White	69 (80.2%)
Black	3 (3.5%)
South Asian	3 (3.5%)
East Asian	3 (3.5%)
Other	8 (9.3%)
Assisted reproductive technology	6 (7%)
MVP at admission (mm)^a^	34 (25.50)
MVP during hospital stay (mm)^b^	24 (17.37)
Last MVP before delivery (mm)^c^	21 (16.28)
Oligohydramnios	60 (71%)

Data are shown as total number (percentage), median (25th–75th interquartile range) or mean (± standard deviation)

*MVP* maximum vertical pocket of amniotic fluid

^a^Data available in 72/86 patients

^b^Data available in 50/86 patients

^c^Data available in 64/86 patients

**Fig. 2:**
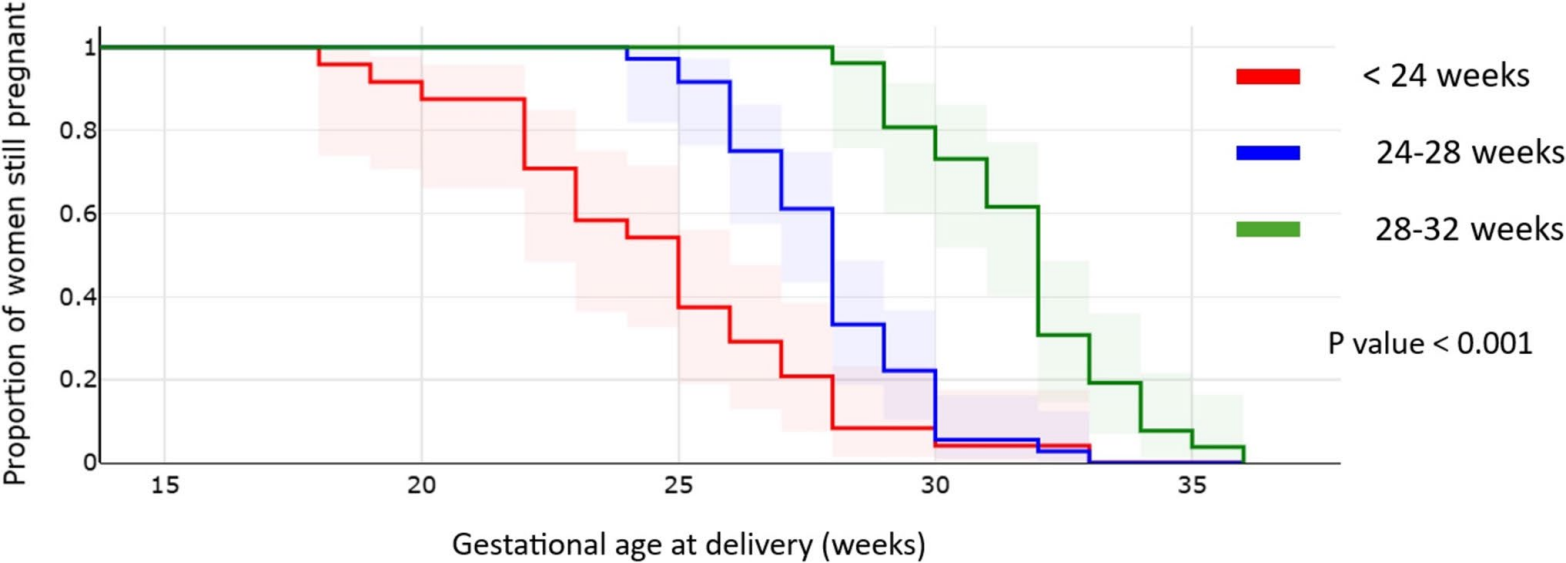
Kaplan Meier curves of probability of continued pregnancy among women with PPROM at different gestational ages

Detailed data regarding the latency from PPROM to delivery are reported in Table 2. Overall, the mean GA at PPROM in the study group was 26.1 weeks and the median latency to delivery was 16 days (interquartile range, IQR, 4–27 days). The median latency to delivery was 22.5 days for PPROM occurring before 24 weeks of gestation, 11 days for PROM occurring between 24 and 28 weeks, and 16 days for PPROM between 28 and 31 + 6 weeks (p = 0.29). Twenty-nine percent of patients with pre-viable PPROM and 35% of those with PPROM between 24 and 32 weeks delivered within one week from membrane rupture. Kaplan Meier curves of probability of continued pregnancy among women with PPROM at different gestational ages are represented in Fig. 2. As shown in Table 2, the livebirth rate and neonatal survival at discharge rate exceeded 90% when PPROM occurred after 24 weeks of gestation. In cases of previable PPROM, the mean gestational age at delivery was 25 weeks and neonatal survival at discharge was 58%, which was significantly lower when compared to survival rates in cases of PPROM occurring between 24 and 25^+6^ weeks (p = 0.041, Table 3) or between 24 and 27^+6^ weeks of gestation (p = 0.002, Table 3).

**Table 2:** Latency from PPROM to delivery by gestational age at PPROM

Gestational age at PPROM (weeks)	n	Gestational age at delivery (weeks)	Latency to delivery (days)	Delivery within 48 h n (%)	Delivery within 7 days n (%)	Livebirth	Neonatal survival at discharge n (%)
All patients (mean GA at PPROM 26.1 weeks)	86	28.6 (± 3.6)	16 (4, 27)	13 (15%)	29 (33.7%)	77 (89.5%)	73 (84.9%)
< 24	24	25.1 (± 3.5)	22.5 (3.75, 35.8)	4 (16%)	7 (29%)	17 (70.8%)	14 (58.3%)
24 + 0–27 + 6	36	28.4(± 1.9)	11.0 (3.0, 21.8))	7 (19.4%)	15 (41.7%)	34 (94.4%)	33 (91.7%)
28 + 0–31 + 6	26	32.1 (± 1.9)	16.0 (6.5, 25.3)	2 (7.7%)	7 (26.9%)	26 (100%)	26 (100%)

Data are shown as total number (percentage), median (25th–75th interquartile range) or mean (± standard deviation)

**Table 3:** Neonatal survival at discharge in cases of previable preterm premature rupture of membranes (pPPROM) compared to PPROM after 24 weeks

Outcome	PPROM < 24 weeks (n = 24)	PPROM 24^+0^–25^+6^ weeks (n = 12)	p-value^a^	PPROM 24^+0^–27^+6^ weeks (n = 36)	p-value^a^
Survival at discharge n (%)	14 (58.3%) 18–20 + 6 weeks: 57.1% 21–22 + 6 weeks: 42.9% 23–23 + 6 weeks: 70%	11 (91.7%)	0.041	33 (91.7%)	0.002

^a^Compared to PPROM < 24 weeks

**Fig. 3:**
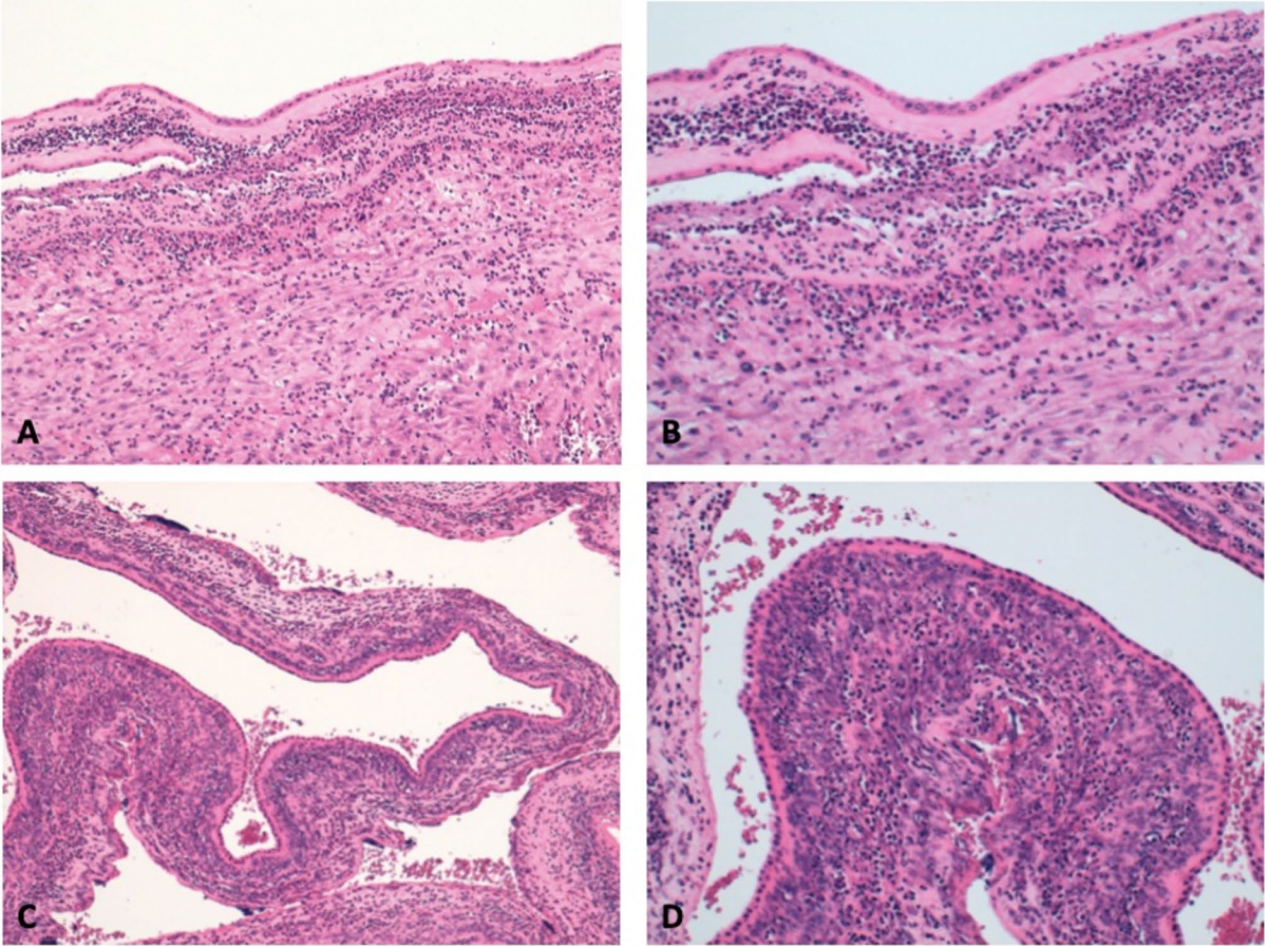
Sections of amniochorionic membranes characterized by chorioamnionitis (H&E **A**, **C** magnification 10×; **B**, **D** magnification 20)

Detailed incidences of the adverse obstetric outcomes categorized by gestational age at PPROM are provided in Table 4. While there was no case of placental abruption or cord prolapse in PPROM occurring after 28 weeks of gestation, the estimated incidences in very early- PPROM cases (< 28 weeks) exceeded 10%. Within this group of PPROM, the rates of cesarean section and histologic chorioamnionitis were also notably high, reaching 64% and 50% of cases, respectively, among cases with PPROM between 24 and 28 weeks. However, when these outcomes were compared between PPROM occurring before 28 weeks and those between 28 and 31^+6^ weeks, the difference was not statistically significant (Table 5). Figure 3 illustrates a photomicrograph of amniochorionic membranes from one of the cases of histologic chorioamnionitis.

**Table 4:** Obstetric outcomes by gestational age at PPROM

Gestational age at PPROM (weeks)	n	Gestational age at delivery (weeks)	Placental abruption n (%)	Cord prolapse n (%)	Cesarean section	Clinical chorionamnionitis	Histologic chorioamnionitis
All (Mean GA at PPROM 26.1 weeks)	86	28.6 (± 3.6)	7 (8.1%)	6 (7%)	43 (50%)	17 (19.8%)	38 (44%)
< 24	24	25.1 (± 3.5)	3 (12.5%)	2 (8.3%)	9 (37.5%)	6 (25%)	10 (41.7%)
24 + 0–27 + 6	36	28.4 (± 1.9)	4 (11.1%)	4 (11.1%)	23 (63.9%)	5 (13.9%)	18 (50%)
28 + 0–31 + 6	26	32.1 (± 1.9)	0	0	11 (42.3%)	6 (23.1%)	10 (38.5%)

Data are shown as total number (percentage), median (25th–75th interquartile range) or mean (± standard deviation)

**Table 5:** Comparison of adverse obstetric outcomes between cases of PPROM occurred below 28 weeks and between 28 and 31^+6^ weeks

	Gestational age at PPROM		p-value
	< 28 weeks (n = 60)	28^+0^ + 0–31^+6^ weeks (n = 26)	
Placental abruption n (%)	7 (11.7%)	0	0.10
Cord prolapse n (%)	6 (10%)	0	0.17
Histologic chorioamnionitis* n (%)	28 (46.7%)	10 (38.5%)	0.48
Cesarean section	32 (53.3%)	11 (42.3%)	0.09

Chi-squared and Fisher’s Exact tests used for categorical data comparison

## Discussion

Our study has shown that the median latency to delivery is 3 weeks for pre-viable PPROM and around 2 weeks for cases of PPROM between 24 and 32 weeks. Additionally, we observed that all cases of placental abruption and cord prolapse occurred in women that had membrane rupture before 28 weeks of gestation, and that neonatal survival at discharge increased by more than 33% when PPROM occurred after 24 weeks of gestation compared to earlier gestational ages. The occurrence of placental abruption was significantly associated with the latency to delivery (median latency 32 days in women with abruption vs 12 days in women without abruption, p = 0.032).

Closer examination of latency at different gestational ages at PPROM in our study group revealed that the median latency tends to be longer in cases of previable PPROM compared to PPROM occurring later, although the difference was not statistically significant. Many previous studies have suggested an increased latency with earlier PPROM [4, 5, 20–22]. In previable PPROM, we observed a median latency of 22 days, which is consistent with the findings by Manuck et al. [23]. On the other hand, in their review of 15 studies on previable PPROM, Sim et al. reported that the median latency ranged between 7 and 49 days, which reflects the heterogeneity between studies [3].

The median latency that we observed among PPROM after 24 weeks (13.5 days for all PPROM occurring between 24 and 31^+6^ weeks) is comparable to that reported in the study by Baser et al. [22], who observed a mean latency of 15 days in the whole study cohort, although they included cases of PPROM up to 35 weeks of gestation. When comparing different groups of gestational age, the median latency observed in the 24–28 weeks PPROM group in our study is similar to the mean latency reported by Baser et al. [22] in the same GA interval. However, in the 24–28 weeks group, they observed a much longer mean latency of 24 days (compared to a median of 11 days in our cohort). Nonetheless, the authors reported the mean value, along with a large standard deviation [22]. Given the non-parametric distribution of the data on latency, we opted for median and IQR, limiting the ability to compare with the results of that study.

Regarding neonatal outcomes, the rate of neonatal survival at discharge in cases of previable PPROM, where the mother delivered at a mean GA of 25 weeks, was 58%, which is higher than that reported by Sim et al. (44.9%) [4] and closer to the data by Manuck et al. [24]. This improvement likely reflects advancement in neonatal intensive care unit techniques. In fact, our data collection covers the last 5 years, while the studies reviewed by Sim et al. included women with PPROM between 1988 and 2014.

In cases of PPROM occurring after 24 weeks, neonatal survival at discharge in our cohort exceeded 90%, and it reached 100% for PPROM after 28 weeks. This survival rate is higher than that reported in a previous study of a cohort of 228 women with PPROM managed in a single center before 2005 [25] and it may be related to differences in obstetric and neonatal care protocols.

In terms of adverse obstetric outcomes, the overall incidence of placental abruption in our cohort was 8%, which is higher than that reported by Major et al. (5%) and Ananth et al. (2%) in women with PPROM [10, 26]. This difference can be attributed to the fact that those studies included all cases of membrane rupture before 37 weeks of gestation. Since our analysis focused on early-, very early-, and previable PPROM, we anticipated a higher incidence of such outcome. Indeed, all cases of placental abruption (7/86) and cord prolapse (6/86) in our cohort occurred in women who experienced PPROM before 28 weeks.

The high incidence of cord prolapse and cesarean section in PPROM that occurred before 28 weeks may be partly attributed to fetal malpresentation and fetal heart rate abnormalities, respectively, which are more common at earlier gestational ages compared to later ones. Although the difference in the incidence of these outcomes between PPROM occurring before and after 28 weeks was not statistically significant, this may be attributed to the limited number of cases.

The overall rate of clinical chorioamnionitis in our cohort, at 20%, is similar to that observed in the study by Baser et al. [21] and higher than that reported in the study by Ramsey et al. [27]. This difference may be explained by the lower mean gestational age at PPROM in our cohort (26.1 weeks) compared to that study (32.4 weeks). It has been previously observed that the incidence of chorioamnionitis increases with decreasing gestational age at PPROM [26]. Differently from other studies, we also reported the rate of chorionamnionitis upon histologic examination, which was more than 2 times higher than the rate of chorionamnionitis suspected clinically. Among cases of PPROM before 28 weeks, the rate of histologic chorioamnionitis was notably high, detected in almost half of the cases in our study.

The strengths of the present study include the relatively large sample size, considering the low incidence of PPROM < 32 weeks (less than 1% of all deliveries in our hospital during the study period), and the fact that all cases were managed in a single tertiary center, with a uniform management based on the hospital’s protocol. The stratification of outcomes based on the timing of PPROM is another strength, as it provides specific information allowing for more personalized counselling for patients regarding maternal and neonatal outcomes.

This study also has some limitations, including its retrospective design, the absence of data on cases of PPROM that opted for elective termination of pregnancy before viability, which represents a common inherent selection bias in studies on PPROM, and the lack of neonatal long-term follow-up data.

## Conclusions

In summary, our findings provide valuable information for patients counselling, reflecting advancements in obstetric and neonatal care compared to older studies, and support the need for careful inpatient management of cases of PPROM once the pregnancy has reached viability, and particularly before 28 weeks, when the incidence of cord prolapse and placental abruption is higher.

## Data Availability

The data that support the findings of this study are available on request from the corresponding author.
